# Advantages of using touch-controlled, minimally invasive implantation technique on soft tissue in the aesthetic zone of maxillary anterior teeth

**DOI:** 10.1097/MD.0000000000040051

**Published:** 2024-12-13

**Authors:** Wenchao Li, Ning Ruan, Yuan Tian, Senhao Li, Damdindorj Boldbaatar, Batbayar Badral

**Affiliations:** a Department of Oral and Maxillofacial Surgery, School of Dentistry, Mongolian National University of Medical Sciences, Ulaanbaatar, Mongolia; b Department of Oral Implantology, Affiliated Hospital of Chifeng University, Inner Mongolia Key Laboratory of Oral Craniomaxillofacial Diseases Research, Chifeng, China; c Department of Nursing, Affiliated Hospital of Chifeng University, Chifeng, China; d Department of Physiology, Mongolian National University of Medical Sciences, Ulaanbaatar, Mongolia.

**Keywords:** cone-beam computed tomography, flapless implant, implant, minimally invasive

## Abstract

Endosseous implant is an ideal treatment option for the treatment of denture defects and omissions. However, how to maintain the perfect gum contour of patients has been an important research topic for stomatologists and has attracted much attention. The objective of this study was to explore the advantages of minimally invasive touch-controlled implantation in maintaining soft tissue morphology in the aesthetic area of maxillary anterior teeth. Forty patients underwent preoperative cone-beam computed tomography and were randomly divided into 2 groups: experimental group (touch-controlled, minimally invasive implantation approach, n = 20) and control group (traditional flap implantation approach, n = 20). The operative time, postoperative pain, gingival recession, and patient satisfaction were compared between the 2 groups. The independent sample *t* test was employed to compare the results, with a significance level of α = 0.05. Statistical significance was determined at *P* < .05. The operative time was 4.50 and 9.21 minutes, respectively, for 2 groups, *P* < .001. The mean pain scores of 2 groups were 1.09 and 2.14, respectively, at 24 hours after surgery, *P* < .001. The H values (the distance from the labial edge of the implant healing cap to the lowest gingival margin), 6 weeks after surgery, of 2 groups were 2.69 and 3.05, respectively, *P* = .023. There was a significant difference in patient satisfaction between 2 groups 24 hours after surgery (*P* < .001). Touch-controlled minimally invasive implantation has shorter operative time and lesser bleeding, postoperative pain, and gingival recession than traditional flap implantation, which is conducive to the preservation of gingival shape.

## 1. Introduction

Dentition defects and loss of dentition are the most common and frequently encountered dental disorders in humans.^[1]^ Implant-supported denture is the most ideal treatment option for fixing such disorders.^[1,2]^ However, not only relieving pain in patients but also preserving their gingival contour has always been an important research topic for stomatologists and has attracted much attention. Since Brånemark proposed the osseointegration theory in 1969,^[3]^ people have greatly deepened the concept of implant. Conventional dental implant surgery requires incision of the alveolar mucosa flap, the preparation of fossa, and the implantation of implants. Although the procedure is standard, postoperative complications such as pain and swelling may cause discomfort to patients.^[4]^ The blood supply to the alveolar bone is mainly from 3 sources: mucoperiosteal blood vessels, vascular plexus of periodontal ligaments, and cancellous intraosseous vessels. After tooth loss, the vascular plexus of the periodontal ligaments disappear, and the blood supply is mainly from the other 2 sources. In this case, opening and turning the flap will lead to insufficient blood supply to the alveolar mucosa vessels. Therefore, the blood supply of the alveolar bone mainly depends on the internal vessels of the cancellous bone. The blood supply to the cortical bone depends on the internal blood vessels of the cancellous bone, which means that there will be a certain degree of soft and hard tissue absorption during the treatment process.^[5,6]^ This phenomenon is likely to affect the aesthetic appeal after implantation.

The concept of flapless implant surgery was first proposed by Kan (2000).^[7]^ Subsequent basic experimental studies and clinical case observations provided the theoretical basis for flapless implantation and confirmed its clinical feasibility and advantages.^[8–12]^ In recent years, due to the advent of cone-beam computed tomography (CBCT) and the improvement in the instruments and equipment available for implants, the cognition levels of surgeons and the operational ability on hard tissues in the oral and maxillofacial regions have improved, thus leading to the possibility of minimally invasive implantation technology.^[13–16]^

Currently, flapless implantation is categorized into 2 distinct methodologies: “surgical guide” and “touch-controlled” implantation.^[8–12]^ These approaches represent a departure from traditional implant surgery practices, with the former emphasizing guided implantation facilitated by computer-aided design. The latter underscores the utilization of the operator’s tactile feedback through auxiliary finger touch to harmonize and regulate manual coordination, thereby achieving a comprehensive minimally invasive implant surgery process. Wu Dayi (2015)^[17]^ posit that the successful implementation of touch-controlled minimally invasive implantation necessitates a robust foundation in multidisciplinary theory and practical operational skills. This technology stands as the cornerstone for the execution of minimally invasive implantation surgeries.

In this study, the primary focus pertained to the operational intricacies and clinical efficacy of the minimally invasive implantation technique. The discourse delved into the procedural nuances and therapeutic outcomes. This investigation aimed to elucidate the procedural protocols and therapeutic impact associated with the utilization of touch-controlled, minimally invasive implantation techniques in clinical settings.

## 2. Materials and methods

### 2.1. Research design

In this clinical trial, a total of 40 patients were selected through random assignment facilitated by a coin toss, then allocated into either the trial group (touch-controlled minimally invasive implantation group) or the control group (traditional flap implantation) following randomization procedures. The study employed a single-blind methodology, ensuring that the subjects were unaware of the specific surgical procedure they received. A total of 40 implants were surgically placed, with 20 implants in each respective group. Osstem TS III implants manufactured by Osstem Implant Co., Ltd. in Seoul, South Korea, were utilized in this investigation. Clinical data encompassing operation duration, soft tissue metrics, and postoperative pain levels were meticulously documented by a proficient oral implantologist.

### 2.2. Patient inclusion and exclusion criteria

#### 2.2.1. Inclusion criteria

1. From January 2021 to June 2023, a study was conducted in the Department of Oral Implantology at the Affiliated Hospital of Chifeng University in Inner Mongolia, China, focusing on patients undergoing dental implant procedures.
2. The age range is 18 to 60.
3. Absence of a single maxillary central incisor tooth without evident bone defects or bone augmentation.
4. Proximal and distal gap dimensions of the missing tooth space falling within 7 to 9 mm, with a minimum available bone width of ≥6.5 mm as measured by CBCT.
5. Presence of healthy adjacent teeth.
6. No systemic diseases impacting implant restoration.
7. Patients who provided written consent for this study.

#### 2.2.2. Exclusion criteria

1. Presence of continuous polydontia.
2. Clearly inclined adjacent teeth and noticeably elongated opposing teeth.
3. Patients diagnosed with moderate to severe periodontitis or progressive periodontitis.
4. Heavy smokers consuming more than 10 cigarettes daily.
5. Individuals undergoing head and neck radiotherapy.
6. Oral mucosal fibrosis.

### 2.3. Sample size confirmed

The sample size formula for a statistical test based on the difference in sample means can be expressed as follows: n1 = n2 = 2[(uα+uβ)s/δ]2 + uα2/4. As per findings in the literature,^[18]^ the pain intensity associated with minimally invasive implantation without a flap differs from that of open flap implantation, with a standard deviation of approximately 0.8, and a mean difference between the 2 groups of δ = 1.4. Considering a significance level of α = 0.05 (two-tailed) and a power of β = 0.1 (one-tailed), it was determined that 12 patients undergoing implantation were necessary in each group. To account for potential data loss and individual data incompleteness, the final sample size was set at 20 patients per group.

### 2.4. Ethical approval and informed consent

This research has obtained ethical approval from the Clinical Research Ethics Committee of the Affiliated Hospital of Chifeng University, Inner Mongolia, China (Ref: fsyy2021 49), and the Ethics Committee of the National Medical University of Mongolia (Ref: 2023/3-04) also granted approval for the study, which has been duly registered with the National Research Clinical Research Information Service. It is important to note that this study adheres to a prospective design, with all methodologies conducted in strict compliance with the principles and regulations outlined in the Declaration of Helsinki. Furthermore, all participating patients provided their informed consent prior to their involvement in the study.

### 2.5. Preparation for operation

Preoperative examinations of CBCT, blood routine, coagulation function, liver and kidney function, infectious disease screening, and tooth cleaning were conducted. Oral antibiotics were given 1 hour before surgery (Cephalexin capsule 0.5 g, produced by Guangdong Huanan Pharmaceutical Group Co. Ltd., Guangdong Province, China; ornidazole dispersible tablet 0.25 g, produced by Tianfang Pharmaceutical Co., Ltd., Guangdong Province, Heinan Province, China). Implant size and healing cap were selected according to CBCT imaging results.

### 2.6. Surgical procedure

#### 2.6.1. Touch-controlled invasive implantation

**Figure 1.**
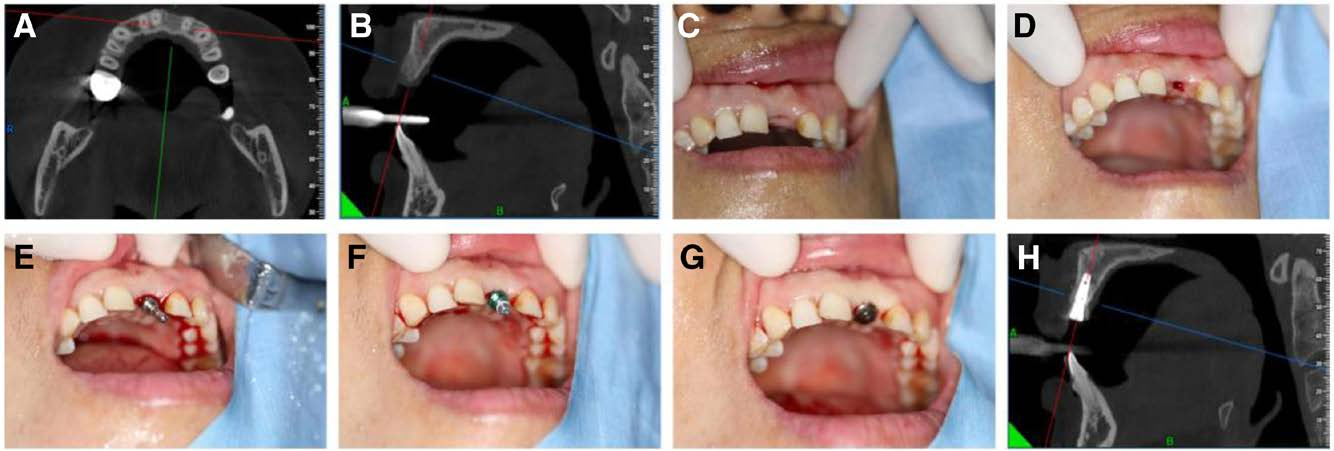
(A) Conical beam computed tomography (CBCT) shows preoperative bone integrity on cross section; (B) conical beam computed tomography (CBCT) showed preoperative bone integrity on sagittal plane; (C) a preoperation picture showing tooth loss in a front view; (D) touch-controlled minimally invasive flapless implant nest preparation; (E) detect the orientation of implant nest preparation; (F) the implant was inserted into the alveolar bone; (G) the implant is fitted with a healing abutment; (H) bone profile with an implant after the surgery.

Conventional disinfection, local anesthesia administration using articaine epinephrine solution (French Blue Company), periodontal probe to determine the implant site. The approach involves quantifying the interproximal and distal measurements of the absent tooth alongside the buccal and lingual aspects utilizing SOWDANE (manufactured by Shanghai Shuoti Technology Co., Ltd., China). The central convergence of these dual measurements designates the location of the implant site. Gingival circumferential incision, step-by-step removal of the “〇” shape of the internal mucoperiosteum at the incision site, and implantation based on the length and diameter of the intended implant to obtain good initial stability of the implant were performed following the aseptic surgical procedures. The implant platform was located 0.5 to 1 mm below the alveolar bone margin and 2 to 3 mm away from the adjacent tooth roots. The healing abutment was installed (Fig. 1), and the permanent repair of the superstructure was completed after 3 months.

#### 2.6.2. Traditional flap implantation

**Figure 2.**
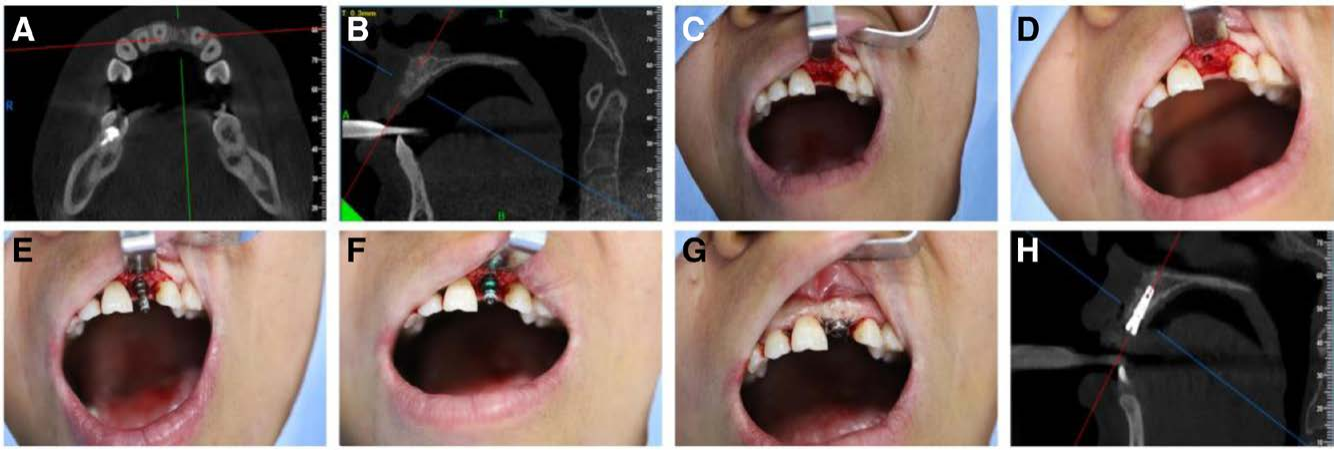
(A) Conical beam computed tomography (CBCT) shows preoperative bone integrity on cross section; (B) conical beam computed tomography (CBCT) showed preoperative bone integrity on sagittal plane; (C) intraoperative photos show tooth loss and gingival incision and flap; (D) traditional flap implant nest preparation; (E) detect the orientation of implant nest preparation; (F) the implant was inserted into the alveolar bone; (G) the implant is fitted with a healing abutment; (H) bone profile with an implant after the surgery.

Conventional disinfection was performed and local anesthesia was administered using articaine epinephrine solution. An incision was made on the partial palatal side of the alveolar crest, and the mucosteal flap was opened to expose the crest of the alveolar bone. Holes were made step by step according to the length of the implants to be implanted, and implants were implanted. All the above steps were performed following the aseptic surgical procedures. The healing abutment was installed at a distance of 2 to 3 mm from the roots of the adjacent teeth, and the gums were trimmed and sutured closely (Fig. 2). The permanent repair of the superstructure was completed after 3 months.

Twenty-four hours after the surgery, antibiotics cephalexin capsule and ornidazole dispersible tablet were prescribed at a dose of 2 g/day and 0.5 g/day, respectively. Gargling with chlorhexidine oral rinse (Shenzhen Nanyang Pharmaceutical Co. Ltd., Shenzhen, China) was prescribed for 1 week at the rate of 3 times a day. The ibuprofen sustained-release capsule (0.6g/day), produced by Tianjin Schix Pharmaceutical Co., Ltd., China, is indicated for oral administration in cases where postoperative pain disrupts sleep patterns. The patients were also advised not to undertake any strenuous exercise for 3 days. Wound healing and pain were examined at 24 and 72 hours after surgery. The degree of gingival recession was examined at 3 and 6 weeks after surgery. After the completion of planting and restoration, a review was conducted once every 3 months for the first year and once every year thereafter.

### 2.7. Evaluation index

#### 2.7.1. General characteristics of patients

The age, sex and dental position of patients were recorded.

#### 2.7.2. Clinical data

**Figure 3.**
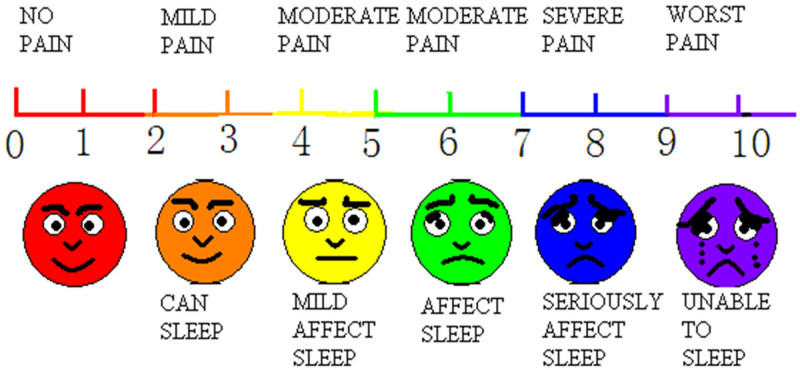
Visual analog scale (VAS).

(1) The operative time of each tooth implantation (from mucosal incision to implant completion) was recorded in the experimental group and the control group, and compared with the operative time of conventional flap implantation (from mucosal incision to implant completion), and the difference was analyzed.
(2) Postoperative pain in patients was evaluated based on the visual analog scale (VAS) scores^[19]^ (Fig. 3). An oral pain reliever was prescribed when the score was above 4.
(3) The degree of gingival recession of both the experimental and the control groups at 3 and 6 weeks after surgery was measured using a Vernier caliper. The distance “H” from the top edge of the labial healing cap to the lowest point of the labial gingival margin was taken as the reference standard and the mean value of each site was measured 3 times, accurate to 0.01 mm.
(4) The VAS score is used for scoring patient satisfaction. The measuring scale is 10 cm in total, and each 1 cm represents 1 point. The left-most point refers to 0 points, meaning highly unsatisfactory, and the right-most point refers to 10 points, meaning highly satisfactory.

### 2.8. Statistical treatment

The statistical analysis in this study was conducted using SPSS 20.0 software (Beijing NetData Times Technology Co., Ltd., Beijing, China). Normality of the data within each group was assessed using the Shapiro–Wilk test for measurement data. Data conforming to a normal distribution was expressed as $\bar{\chi }\pm s$. Group comparisons were performed using the independent sample *t* test. The mean between the 2 groups that did not conform to the normal distribution was tested by Wilcoxon rank sum test (α = 0.05).

## 3. Results

### 3.1. General characteristics of patients

The research encompassed a cohort of 40 individuals who received maxillary central incisor implants across 40 implantation sites. The cohort comprised 19 males (47.50%) and 21 females (52.50%), with ages ranging from 18 to 60 years, having an average age of 36.33 ± 9.04 years (Table 1).

**Table 1:** General characteristics of patients.

Groups	Cases	Gender		Age $\bar{\chi }\pm s$	11 loss	21 loss
		Male	Female			
Experimental group	20	9	11	34.22 ± 10.03	8	12
Control group	20	10	10	38.43 ± 8.05	11	9

### 3.2. Operation time

T test was conducted on the operative time of both the experimental and the control groups. The results showed the operative time of the experimental group to be 4.50 ± 0.56 minutes and that of the control group to be 9.21 ± 1.52 minutes; t = 10.22 and *P* = .000. It may be observed that the operative time of the experimental group is significantly shorter than that of the control group, and that the difference is significant.

### 3.3. Pain status measurement

The pain status of the patients was evaluated 24 and 72 hours after the surgery using the self-assessment criteria. The results of the statistical analysis are shown in Table 2. As can be seen from the table, the short-term postoperative pain degree of the experimental group was significantly lower than that of the control group.

**Table 2:** Pain status of patients 24 and 72 hours after surgery.

Time	n	Experimental group	Control group	*P*-value
24 hours after surgery	20	1.09 ± 1.04	2.14 ± 1.76	<.001
72 hours after surgery	20	1.04 ± 0.64	1.28 ± 0.72	.581

### 3.4. Gingival recession measurement

The gingival recession of both the experimental and the control groups was measured 3 and 6 weeks after surgery, and the data were analyzed statistically. The results are shown in Table 3. There was no significant difference in the gingival recession of the 2 groups 3 weeks after implantation, but there were differences 6 weeks after implantation; it may be observed that the gingival recession of the experimental group was significantly lower than that of the control group, with statistical significance.

**Table 3:** Gingival recession in patients from both experimental and control groups, 3 and 6 weeks after surgery.

Time	n	Experimental group	Control group	*P*
3 weeks after surgery	20	2.28 ± 0.12	2.69 ± 0.11	.265
6 weeks after surgery	20	2.38 ± 0.14	3.05 ± 0.10	.023

### 3.5. Patient satisfaction score

The satisfaction scores of the experimental and control groups obtained at 24 and 72 hours after the operation are shown in Table 4. As can be seen from the table, patient satisfaction was significantly higher in the experimental group than in the control group 24 hours after surgery, and the difference was statistically significant.

**Table 4:** Comparison of the VAS scores between the 2 groups.

Time	n	Experimental group	Control group	*P*
Time of operation	20	8.78 ± 1.13	8.47 ± 0.98	1.032
24 hours after surgery	20	9.00 ± 0.84	7.97 ± 1.15	.000
72 hours after surgery	20	9.08 ± 0.88	8.92 ± 0.90	1.651

## 4. Discussion

With continuous developments in oral implantology, minimally invasive and simple techniques have become a trend in dental implantology. With the improvement in implant surface treatment technology and the development of modern medical imaging,^[13–15,18–21]^ minimally invasive implantology is all set to become the future direction of oral implantology. All the patients in this study had CBCT taken before the surgery to obtain the available length, width, and height of the alveolar bone at the implant site, analyze the bone mass, and determine the anatomical structure around the implant site. The aseptic surgical procedure was strictly applied during the surgery. The difference was that the experimental group adopted minimally invasive non-flap surgery by hand control, while the control group adopted conventional open flap surgery. Touch-controlled, minimally invasive implantation surgery reduced intraoperative and postoperative bleeding and saved operative time because it did not require incision and suture. The observation was similar to the research results of many scholars.^[21–23]^ However, since touch -controlled, minimally invasive implantation cannot be performed under direct vision, more time is required for preoperative evaluation and design.

From the perspective of the postoperative pain degree of patients, there was a significant statistical difference in the scores of the experimental and control groups 24 hours after surgery, indicating that the pain response after minimally invasive implantation was relatively mild; the observation was similar to the results in a study by Fortin (2006).^[24]^ The main reason for the decreased pain degree in patients of the experimental group was that the mucopleural tissue flap was not opened in the minimally invasive implantation surgery, and the soft tissue injury was small. There was no local edema or hematoma after the surgery and the mucosal tissue healed quickly. The success of the implantation depends on the existence of the stable soft tissue barrier around the implant, which can protect the bone tissue and maintain the aesthetics and function of the gingiva, especially the attached gingiva around the implant, which can not only prevent gingiva retraction and provide a tight cuff around the implant but also help patients maintain good oral hygiene and prevent the occurrence of peri-implant inflammation.^[25,26]^ In this study, the gingival recession degree of the experimental and control groups was compared 3 and 6 weeks after surgery. No significant statistical difference in the “H” value between the 2 groups was observed at 3 weeks post-surgery. However, by the sixth week, this value showed a significant difference, and the “H” value of the experimental group was significantly lower than that of the control group. Kim (2009)^[27]^ found in an experimental study on dogs that the gingival blood flow in non-flap surgery was much better than that in flap surgery, which was beneficial to mucosal tissue healing and reduced gingival retreat and bone resorption. The observation also confirms the findings of this study that minimally invasive implantation not only helps maintain the shape of the gums but also reduces gum receding.

VAS was also used to evaluate patient satisfaction. There was no difference in the satisfaction scores between the experimental and control groups at the time of surgery and 72 hours after surgery. However, the VAS score of the experimental group was found to be significantly higher than that of the control group 24 hours after the surgery. The reason may be that, after the wearing off of anesthesia, the pain degree of the experimental group is much less than that of the control group, which reduces the satisfaction of the patients in the control group with the surgical treatment.

Although touch-controlled, minimally invasive implantation offers numerous advantages, the associated risks are relatively significant. Strict adherence to appropriate patient selection criteria is crucial to ensuring the success of touch-controlled, minimally invasive implantation procedures. Uneven alveolar ridges or thin bone walls increase the likelihood of partial implant exposure. Optimal preservation of lip and lingual bone wall integrity necessitates preparing the implant site on a relatively flat or thick alveolar ridge. Therefore, the alveolar crest must be flat and have a minimum thickness of 6.5 mm. Given the pivotal role of bone density in initial implant stability, preimplantation CT imaging is typically necessary to assess bone density accurately. Furthermore, in minimally invasive guided implantation, thicker mucous membranes are preferred, with a thick gingival biotype offering optimal outcomes in terms of aesthetics and initial postimplantation stability.^[28]^ Touch-controlled, minimally invasive implantation becomes unfeasible if bone mass is inadequate or if bone defects necessitate bone grafting or artificial bone augmentation during the procedure.

Although minimally invasive implantation has many advantages, the touch controlled method adopted in the study puts forward higher requirements from clinical implant surgeons. First, touch-controlled, minimally invasive implantation diagnosis and treatment plan require the correct identification of the CBCT images and accurate evaluation of bone mass, shape, and volume.^[29–31]^ Usually, type 2 and type 3 bones do not change the clinical treatment plan of doctors; however, type 4 bone has a greater impact than other types. Second, since the implantation technique is a dental implant surgery guided by a nonsurgical guide, it requires the implant surgeons to have rich surgical experience and mastery of the standard operating procedure.^[17]^ The standard operating procedure of minimally invasive implantation with touch controlled includes the following: (1) Surgical position. The positioning is done according to the standard postures of implant surgery.^[17]^ (2) Location of implant sites. The periodontal probe was used to accurately locate the implant site. (3) Finger sensation. The surgeon touches the lateral mucosa of the alveolar ridge (buccal, lip, tongue, and palatal) in the planting area with the finger abdomen of the thumb, index finger, and middle finger of the auxiliary hand to feel the subtle movement of the implant drill in the bone and to know whether the drill vibrates and squeeches the bone wall during the cutting process. At the same time, the operator uses the mobile phone and the implant drill with the auxiliary hand to find out the best implant site and reaming direction. It is important to avoid alveolar ridge bone wall perforation and damage. (4) Stable fulcrum. It is important to keep the fulcrum of the operator’s hand stable to prevent the hand-held mobile phone from shaking or moving, which may affect the preparation process of the planting nest. (5) Coordination of bone extrusion. The 4 different types of bones were prepared by using 2.0 reaming drill holes, following which the bone extruder was used in the set direction for extrusion and implantation.

Based on the principle of biologic width, the pivotal factor influencing the aesthetic outcome of dental implants lies in maintaining optimal gingival height. Insufficient lip and buccal gingival height in implanted teeth can lead to incongruities in gingival levels between the implants and neighboring natural teeth, thereby affecting the harmony in clinical crown lengths and overall aesthetic appeal. Thus, this investigation primarily examines the extent of gingival recession on the labial aspect of the implant subsequent to touch-controlled minimally invasive implantation. Recent research indicates that implants inserted using minimally invasive flapless techniques exhibit reduced labiolingual bone resorption,^[32,33]^ with the surrounding alveolar bone serving as crucial support for the gingiva. In cases where bone resorption occurs around the implant, insufficient gum and gingival papilla height may ensue, manifesting as the dreaded “black triangle” phenomenon that detrimentally impacts aesthetics, particularly in the anterior dental region. Consequently, the alveolar bone and gingival height around the implant emerge as pivotal determinants of implant aesthetics. Subsequent investigations will also delve into the alterations within bone tissue surrounding implants post touch-controlled minimally invasive implantation.

## 5. Conclusions

Touch-controlled minimally invasive implantation offers advantages such as reduced operating time, minimized blood loss, decreased postoperative discomfort, and limited gum recession, thereby facilitating gum shape preservation and enhancing patient satisfaction. This surgical approach represents a favorable technique for dental implant procedures, particularly in the aesthetically sensitive anterior dental region, and exhibits potential for extensive clinical adoption.

## Author contributions

**Conceptualization:** Wenchao Li, Ning Ruan, Senhao Li, Batbayar Badral.

**Data curation:** Damdindorj Boldbaatar, Batbayar Badral.

**Formal analysis:** Wenchao Li, Yuan Tian, Damdindorj Boldbaatar, Batbayar Badral.

**Funding acquisition:** Ning Ruan, Damdindorj Boldbaatar.

**Investigation:** Wenchao Li, Batbayar Badral.

**Methodology:** Ning Ruan, Senhao Li, Damdindorj Boldbaatar.

**Project administration:** Yuan Tian.

**Resources:** Senhao Li, Damdindorj Boldbaatar.

**Software:** Ning Ruan, Yuan Tian, Batbayar Badral.

**Supervision:** Wenchao Li.

**Writing – original draft:** Wenchao Li, Yuan Tian, Senhao Li, Damdindorj Boldbaatar, Batbayar Badral.

**Writing – review & editing:** Wenchao Li, Ning Ruan, Yuan Tian, Senhao Li, Damdindorj Boldbaatar, Batbayar Badral.
